# Improved Glycemic Control during a One-Week Adventure Camp in Adolescents with Type 1 Diabetes—The DIACAMP Study

**DOI:** 10.3390/bios14090451

**Published:** 2024-09-21

**Authors:** Antonia-Therese Kietaibl, Faisal Aziz, Eva Wurm, Celine Tomka, Elke Fröhlich-Reiterer, Othmar Moser, Thomas R. Pieber, Peter Fasching, Julia K. Mader, Harald Sourij, Felix Aberer

**Affiliations:** 1 Department of 5th Internal Medicine with Endocrinology, Rheumatology and Gerontology, Clinic Ottakring, 1160 Vienna, Austria; antonia-therese.kietaibl@gesundheitsverbund.at (A.-T.K.); peter.fasching@gesundheitsverbund.at (P.F.); 2 Division of Endocrinology and Diabetology, Medical University of Graz, 8036 Graz, Austria; faisal.aziz@medunigraz.at (F.A.); celine.tomka@stud.medunigraz.at (C.T.); othmar.moser@medunigraz.at (O.M.); thomas.pieber@medunigraz.at (T.R.P.); felix.aberer@medunigraz.at (F.A.); 3 Division of Internal Medicine, Diakonissen Hospital Schladming, 8970 Schladming, Austria; eva.wurm@gmx.at; 4 Department of Pediatrics and Adolescent Medicine, Medical University of Graz, 8036 Graz, Austria; elke.froehlich-reiterer@medunigraz.at; 5 Division Exercise Physiology and Metabolism, Institute of Sport Science, University of Bayreuth, 95444 Bayreuth, Germany; 6 Interdisciplinary Metabolic Medicine Trials Unit, Medical University of Graz, 8036 Graz, Austria

**Keywords:** diabetes mellitus type 1, continuous glucose monitoring, diabetes camp

## Abstract

Adolescence remains a crucial age associated with diabetes distress in individuals living with type 1 diabetes (T1D). The Austrian organization “Diabär” regularly hosts a one-week adventure camp for adolescents (12–18 years) living with T1D. The camp focuses on “fun activities” without a structured educational protocol in order to minimize diabetes distress and increase diabetes management skills. In contrast to educational camps, training is kept to a minimum. However, attendees analyze the glycemic data of the previous day with their medical supervisor once daily during the camp. All subjects used a standardized real-time continuous glucose monitoring (CGM) system (DexcomG7) throughout the whole study. Glycemic metrics were prospectively analyzed during three periods: week 1 = home phase, week 2 = adventure camp, and week 3 = after the camp. Safety (time below range 1 [TBR1], 69–54 mg/dL, and time below range 2 [TBR2], <54 mg/dL) and efficacy (time in range [TIR], 70–180 mg/dL) were assessed by comparing the CGM data during weeks 1–3. The CGM data of 14 participants were analyzed. The TIR was higher during the camp week versus week 1 (70.4 ± 11.1% vs. 53.1 ± 20.2%; *p* = 0.001). The TBR1 significantly increased during camp compared to week 1 (2.5 ±1.7% vs. 1.3 ± 1.2%; *p* = 0.009), whereas the TBR2 did not differ. No serious adverse events occurred. This adventure camp without a main focus on education showed feasibility and safety in adolescents with T1D.

## 1. Introduction

Type 1 diabetes (T1D) represents a chronic autoimmune disease demanding continuous efforts and adherence to blood glucose measurements and/or continuous glucose monitoring (CGM), insulin dosing, and carbohydrate counting in order to maintain glycemic control [1,2,3]. Diabetes management in unfamiliar situations is challenging despite the improvement in diabetes technology. Adolescence remains a crucial age associated with diabetes distress and negative impact on glycemic control. Glycemic challenges like physical activity, vacation, school trips, or parties are omnipresent during pubertal development and may deteriorate diabetes management, reduce disease acceptance, decrease the adoption of independent diabetes self-management skills, and increase diabetes distress with a negative impact on glycemia and mental health in youth and adolescents living with T1D [4]. The era of modern diabetes management provides diabetes technology such as CGM systems and sensor-augmented insulin therapy (continuous subcutaneous insulin infusion [CSII] or automated insulin delivery [AID]) and has demonstrated beneficial effects in glucose control and quality of life for people living with diabetes [5,6]. Non-invasive glucose-measuring methods (optical and electrochemical) other than subcutaneous CGM systems are being researched but do not yet play a widespread role in the daily routine of diabetes management due to various challenges [7,8,9,10]. Despite all the advantages associated with the use of modern diabetes technology, with the visibility of technological devices for diabetes, new stigma challenges faced by the affected children and adolescents have emerged [11]. In reality, parents or caregivers are often responsible for their offsprings’ diabetes control, although adolescents should continuously be encouraged to become independent with regard to their diabetes management [3,4]. Diabetes camps for children and adolescents can help them achieve this independence.

The International Society for Pediatric and Adolescent Diabetes (ISPAD) recommends an interdisciplinary team and a prespecified plan in order to ensure safety at diabetes camps [3]. Most of the existing evidence on diabetes camps for people living with T1D covers structured conditions emphasizing glycemic outcome metrics [12,13] or self-reported impacts on psychological factors [14,15] achieved by a structured intervention protocol including daily training sessions and a detailed documentation of physical activity, meals, and insulin dose. As opposed to existing evidence on diabetes camps for youth and adolescents living with T1D [12,13,16,17,18,19,20], we organized a one-week adventure camp for adolescents living with T1D without parental guidance, where fun activities including a lot of physical activity, comprising swimming, hiking, or visiting climbing parks, were the focus. The central purpose of this camp was to connect the adolescent attendees and promote self-esteem and competence in diabetes management in challenging situations on one’s own. Diabetes education and training were kept to a minimum at this adventure camp. This allowed a reduction in the supervisors required for medical assistance at the camp and might have also reduced diabetes distress by less intensely addressing glycemic management and forcing attendees to continuously confront with their disease.

Therefore, the DIACAMP study prospectively analyzed the safety, efficacy, and sustainability of the glycemic outcomes of an adventure camp for adolescents living with T1D without a specific focus on rigorous and structured diabetes education or diabetes therapy adjustments.

## 2. Materials and Methods

This prospective observational study evaluated glycemic metrics assessed by standardized CGM among unaccompanied adolescents (12–18 years) living with T1D attending an adventure camp in the Austrian alps (sea level of the camp venue, 1.720 m) during three weeks: one week before the camp (week 1), during the one-week summer adventure camp (week 2), and one week after the camp (week 3).

### 2.1. Study Methods

During weeks 1 and 3 (home phases), the participants were not restricted in performing any kind of physical activity and were advised to enjoy their usual (summer holiday) routine. To assess the safety (time below range 1 [TBR1 69–54 mg/dL] and time below range 2 [TBR2 < 54 mg/dL]) and efficacy (time in range [TIR 70–180 mg/dL]) of the adventure camp, we compared the study’s CGM metrics before (week 1), during (week 2), and after (week 3) the camp. The authors decided against an analysis of time in a tight range (70–140 mg/dL), as there are no official ISPAD recommendations for its use in this age group yet [2].

**Figure 1:**
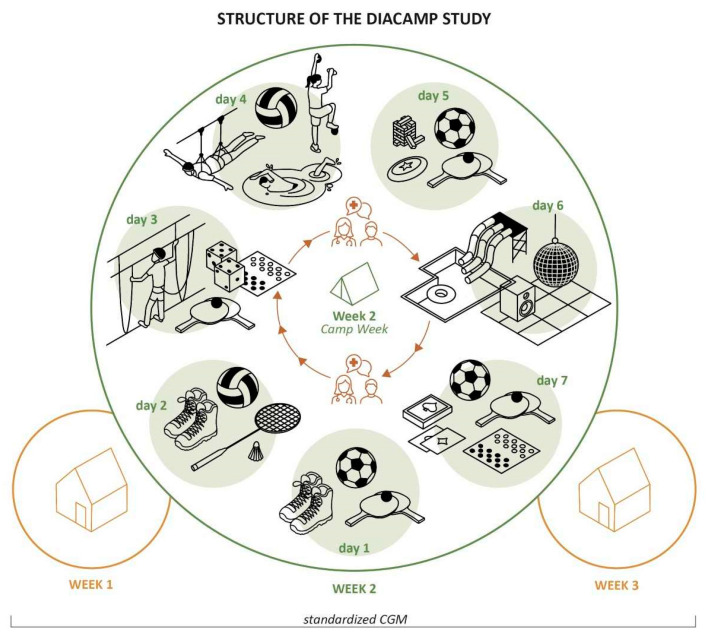
Program of activities during the adventure camp and trial design. Day 1: Get-to-know games, hiking, soccer, table tennis; Day 2: Hiking, ball games, badminton, teambuilding; Day 3: Bird show, hiking park, board games; Day 4: Swimming, volleyball, trampoline, fun park with flying fox; Day 5: Soccer, table tennis, frisbee, coordination games; Day 6: Spa day + water adventure park, disco; Day 7: Indoor soccer, board and card games, farewell; CGM, continuous glucose monitoring.

In contrast to usual educational diabetes camps for youth and adolescents living with T1D, no substantial focus was put on diabetes management (such as documentation of meals, carbohydrate counting, or structured training units) during the adventure camp to meet the intended characteristics of a unique adventure camp: The main focus was to promote the independent and self-responsible attitude toward an individual diabetes management by interacting with other young people living with T1D and acquiring diabetes knowledge from one another. The adventure camp aimed to strengthen the participants’ self-confidence in potential unfamiliar situations such as higher sea level and unusual activities (hiking park, swimming, adventure activities) supervised by the medical staff. The attendees only received an informal conversation with the medical supervisor once daily for about 10 min to discuss potential therapy modifications by evaluating CGM data of the previous day. As diabetes education was not the primary goal of the camp, only two medical doctors for 17 adolescents living with T1D supervised the camp, completing a staff team of five guides including one medical student and two educators, which is quite a low number of involved staff than usual diabetes camps require. The specific camp activities and the trial design are illustrated in Figure 1. The activities lasted between one (table tennis, ball games, badminton, board games, swimming, volleyball, trampoline, soccer, frisbee) and four (hiking, spa day and water adventure park) hours twice a day.

### 2.2. Ethical Considerations

The study was approved by the ethics committee of the Medical University of Graz (EC number: 35-330 ex22/23). The study participants as well as caregivers consented in take part in the study prior to any study-related activities by signing an informed consent (participants and caregivers).

### 2.3. Statistics

Descriptive characteristics for demographic and clinical data were presented by frequency and mean (±standard deviation, [SD]). The significance of each CGM metric was tested with repeated measures ANOVA followed by Bonferroni correction for pairwise comparison of three study periods (week 1 vs. week 2 vs. week 3). A two-sided *p*-value of <0.05 was accepted as significant. The statistical analysis was performed in the R-studio (version 2023.06.0 + 421) and Microsoft Excel.

### 2.4. Outcome Parameters

The primary study endpoints were safety of the camp (defined by CGM-derived TBR1 and TBR2) and efficacy (defined by TIR) during the adventure camp compared to the week prior (week 1) and after the camp (week 3). As a secondary endpoint, the sustainability of the peer-group effect and short education procedures during the camp were assessed by comparing the metrics in the week prior (week 1) and after the camp (week 3). In addition, glycemic control during the day and night was compared between the three study periods (TBR, TIR). Furthermore, a secondary analysis comparing the insulin therapy methods (multiple daily injections [MDI]/AID/CSII) was performed.

### 2.5. Materials 

During the study, all participants followed their usual diabetes management including CGM as well as insulin therapy with MDI, CSII or AID. All participants, not using a Dexcom G7 CGM in their daily routine, were equipped with a blinded Dexcom G7 system for CGM data collection in order to gather homogenous data. Dexcom G7 was used as study CGM because its use is common in Austria, and the separate readers are very usable for the remote monitoring of participants (especially during the night). Capillary glucose measurements were not mandatorily required unless sensors failed or calibration measurements were necessary. All participants used a Dexcom sensor during the entire study. Those who did not use a Dexcom sensor during the daily routine prior to the study were equipped with a sufficient amount of Dexcom G7 sensors and received a training on the system. They used it in a blinded mode in parallel to their actually used system during the three weeks of the study. CGM data were downloaded after three weeks via the Dexcom Clarity software (Version 3.46). Individual sensor data availability of 70% was required for inclusion in the analysis. 

## 3. Results

Seventeen adolescents attended the adventure camp of which 14 (6 females, age 13 ± 2 years, hemoglobin A1c (HbA1c) 7.5 ± 0.9% [58.5± 13 mmol/mol], diabetes duration 6.4 ± 4.4 years, 3 on MDI therapy, 5 on CSII therapy with open loop and 6 on AID-systems) participated in the study. Baseline characteristics are shown in Table 1. A required sensor data availability of >70% was attained by every participant of the study with a mean sensor data availability of 94 ± 6%. All participants completed the study according to the protocol.

### 3.1. Primary Endpoints

#### 3.1.1. Time below Range 

**Figure 2:**
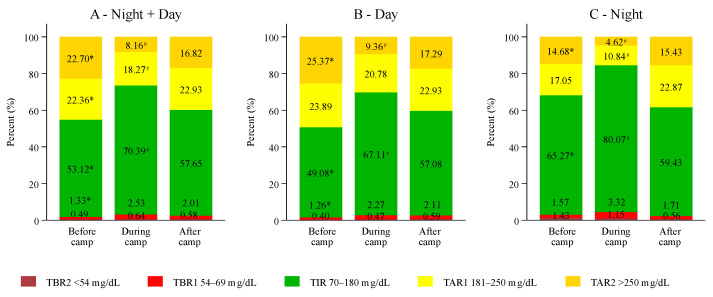
Time in range (TIR), Time below range 1 + 2 (TBR1 + 2), Time above range 1 + 2 (TAR1 + 2) for each study period; (**A**) indicates total data, (**B**) nighttime (10 pm to 8 am), (**C**) daytime (8 am to 10 pm). No significant differences were seen when comparing metrics before vs. after camp. * corresponds to a significant *p*-value for comparison before and during the camp; # corresponds to a significant *p*-value for comparison between during and after the camp.

**Figure 3:**
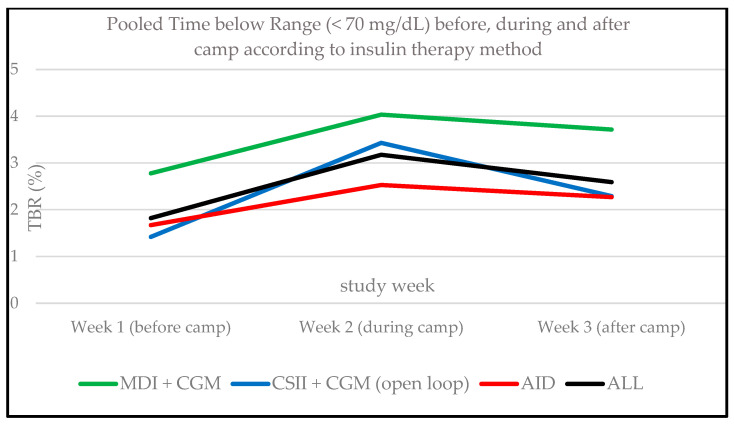
Time below range (TBR < 70 mg/dL) for each study week according to insulin therapy method. y- axis: TBR, x- axis: study week. MDI, multiple daily injections; CGM, continuous glucose monitoring; CSII, continuous subcutaneous insulin infusion; AID, automated insulin delivery. The lines show the pooled participants according to insulin therapy method. ALL represents the graph for the total population.

The TBR1 (69–54 mg/dL) significantly increased during the camp compared to the week before the camp (1.3 ± 1.2% vs. 2.5 ± 1.7%; *p* = 0.009). Level 2 hypoglycemia (TBR2, <54 mg/dL) did not significantly differ between the observation periods (0.5 ± 0.8% vs. 0.6 ± 0.5%, *p* = 0.694; see Figure 2). No episodes of severe hypoglycemia [21] associated with altered mental and/or physical status (level 3 hypoglycemia) requiring medical assistance occurred during the three study weeks. In general, no serious adverse events occurred during all three study weeks. The changes in TBR of each study period are shown in Figure 2 and Supplementary Table S1 and for every attendee according to the insulin therapy method in Figure 3.

#### 3.1.2. Time in Range 

**Figure 4:**
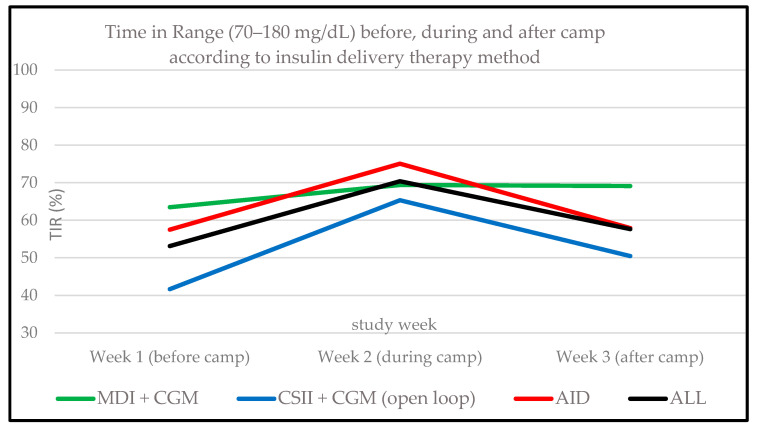
Time in range (TIR 70–180 mg/dL) for each study week according to insulin therapy method; y-axis: TIR, x-axis: study week. MDIs, multiple daily injections; CGM, continuous glucose monitoring; CSII, continuous subcutaneous insulin infusion; AID, automated insulin delivery. The lines show the pooled participants according to the insulin therapy method. ALL represents the graph for the total population.

Time in range (70–180 mg/dL) significantly increased from 53.1 ± 20.2% in the week before to 70.4 ± 11.1% during the camp (*p* = 0.001). Accordingly, time above range (TAR1; >180–250 mg/dL) was reduced during the camp when compared to the week prior to the camp (18.3 ± 6.3% vs. 22.4 ± 4.6%, *p* = 0.043) and TAR2 (>250 mg/dL) decreased (8.2 ± 6.2% vs. 22.7 ± 16.5%, *p* < 0.001) accordingly. While only four participants achieved the recommended target of TIR > 70% [2] in the week before the camp, eight participants reached this goal in the camp week. The summarized data of all individuals (*n* = 14) are shown in Figure 2 and for each participant according to the insulin therapy method in Figure 4. The glucose management indicator (GMI) changed from 7.8 ± 1.0% before to 7.0 ± 0.4% during and 7.5 ± 0.7% after the camp (*p* = 0.001). Glycemia in predefined ranges and further parameters of glycemia according to the study weeks are shown in Supplementary Table S1.

### 3.2. Secondary Endpoints 

#### 3.2.1. Sustainability 

In order to assess whether the peer-group effect and the little extensive talks about glycemia impacted sustainability, metrics were compared between the camp week and week 3. TIR decreased in the week after the camp compared to the camp week (70.4 ± 11.1% vs. 57.7 ± 12.8%, *p* = 0.016) and, in fact, returned to the baseline level observed during week 1, indicating that TIR before and after the camp did not significantly differ. TBR1 and TBR2 did not significantly change between camp weeks 1 and 3. Further data relating to sustainability are presented in Supplementary Table S1.

#### 3.2.2. Comparison of Night Versus Daytime

TIR overnight increased significantly during the camp compared to TIR before and after the camp (80.1 ± 15.5% vs. 65.3 ± 24.4% vs. 59.4 ± 20.5%, *p* = 0.002), while nocturnal hypoglycemia did not differ between the three study weeks and was generally low throughout all three study weeks (TBR1 1.5 ± 1.9% vs. 3.3 ± 3.4% vs. 1.7 ± 1.4% *p* = 0.084 and TBR2 1.4 ± 4.8% vs. 1.2 ± 1.3% vs. 0.6 ± 1.1%, *p* = 0.691). Further differences in day versus night are illustrated in Figure 2 and Supplementary Table S1.

#### 3.2.3. CGM Metrics According to Insulin Therapy Method

TBR (TBR1 + TBR2) was numerically the highest in participants with MDI therapy (*n* = 3) during all three study weeks. TIR was the lowest in those using open loop systems (*n* = 5). Due to the small sample size, no statistical subgroup comparisons were conducted. Pooled TBR/TIR percentage according to insulin therapy method are shown in Figure 3 and Figure 4. Detailed data according to time in different glycemic ranges are shown in Supplementary Table S2.

## 4. Discussion

The prospective DIACAMP study, a one-week adventure camp for unaccompanied adolescents living with T1D, demonstrated safety (TBR1 and TBR2 < 5%) and efficacy (TIR > 70%) as recommended by international guidelines [2] when the pooled data of all participants were used. Although TBR1 significantly increased during the camp week compared to the weeks prior and after the camp, the consensus target for hypoglycemia (TBR1 < 4%) was met continuously, and no severe hypoglycemic episodes occurred despite potentially unfamiliar altitude and exhausting physical activities given that altered daily habituations during the camp might have negatively impacted the risk of hypoglycemic events.

There are no comparable examples for data obtained from adventure camps in youth living with T1D. Several publications report on the impact and importance of high attitude on diabetes management: one case report is covering the personal experience; however, none of them had a similar age group or adventure camp setting [22,23,24], underlining the novelty and importance of our study.

Most of the existing data on diabetes (summer) camps focus on educational settings with a structured camp protocol [12,13,16,17,18,19,20]. In these studies, diabetes camps already demonstrated effectiveness [20], which has been reported as a decrease in HbA1c [25] and positive impact on quality of life [14,26,27]. For example, a recently published study comprising retrospective data of an education camp designed for children with T1D (*n* = 26) revealed an improvement in TIR from 58.2% to 67.0% when the CGM data were compared 2 weeks prior with data gained during the 2-week camp. Similar to our study, this study reports a significant increase in TBR (from 3.2% at baseline to 5.5% during the camp), which was more pronounced during nighttime (from 3.9% to 7.6%) [12]. Of note, in our study, the separate analysis for nighttime did not reveal a significant increase in TBR when periods of and outside the camp were compared.

Glycemia, given as TIR, significantly improved during the camp compared to two reference periods prior and after the camp, although this specific adventure camp mainly aimed to strengthen the self-esteem and- responsibility of adolescents living with T1D, in particular by facing diabetes in a peer group of chronically diseased individuals at a critical age rather than prioritizing diabetes education and training. In detail, eight of the participants achieved a TIR > 70% during the camp, while only four achieved this threshold prior to the camp. Except for two participants, all experienced an increase in TIR during the camp compared to the week before the camp, which underlines the effectiveness of the intervention being restricted to unstructured, daily conversations of a short duration on glucose control with a physician. As the purpose of the adventure camp was to give participants the chance to handle challenging situations including a lot of physical activity with a minimum of intervention by the accompanying adults, it appears that single individuals had difficulties coping with this environment or were not used to regular exercise, leading to a moderate decline in TIR over this short period of time. However, the vast majority (86%) mastered the challenges while even improving TIR. Considering these results, together with the repeated talks on diabetes management, increased physical activity during the camp, potentially alternative diet options provided, and increased adherence to medication could have beneficially influenced the TIR.

However, the glycemic improvement was not sustainable during the week after the camp. TIR as well as TBR were almost identical prior to and after the camp. This could be due to several reasons such as participants being exhausted after the camp performing less than usual exercise, duration of the intervention or the lack of structed education and training sessions during the camp. Moreover, the DIACAMP study took place during school vacations where there might be a major variability of everyday activities and conditions, which could have had a potential influence. While there is a sufficient body of evidence available that diabetes camps positively influence short- and long-term psychosocial outcomes [15,26], rare data exist on whether diabetes camps in general are effective in improving long-term glucose control, especially in the era of modern diabetes technology. Therefore, longer follow-up periods beyond one week might have better determined the true glycemic impact of such an adventure camp. Still, one major aim of this adventure remains feeling comfortable and less stressed with challenging situations for their glucose metabolism.

The major strengths of the DIACAMP study are the prospective design and the use of a unified CGM system for homogenous data analysis. The study cohort reflects a potentially challenging age group, yet we report no drop-outs.

Some limitations have to be announced. The small sample size and lack of standardized evaluation of psychological measures of both adolescents and caregivers have to be reported as limitations of the study. However, existing evidence already demonstrates improved quality of life, treatment satisfaction and reduced diabetes distress measures in adolescents living with T1D attending diabetes camps [25,26,27,28,29]. Future studies should further address this topic by evaluating patient-reported outcome measures and diabetes distress scale data in adolescents living with T1D and their caregivers. Another limitation is that we did not trace the grams of carbohydrate consumed to treat hypoglycemia or data on meal consumption at all, which might provide additive value in order to assess quantitative and qualitative altered nutrition patterns. However, we emphasize that in this special setting of an adventure camp, all disturbing factors such as documentation, carb counting, and capillary measurements were avoided as far as possible in order to stick to the rationale of this depicted camp. The analysis of the different insulin therapy methods must be interpreted with caution due to the small subgroup size and is therefore not generalizable. Moreover, all participants had overall sufficient diabetes knowledge, which might limit the applicability of the results in a general population of adolescents with T1D during a depicted camp.

## 5. Conclusions

The authors emphasize that the DIACAMP study was not a typical educational summer camp, because diabetes-specific training was kept to a minimum, and time-intensive documentation of carbohydrates, insulin doses and physical activity was not required. Despite that, safety and efficacy could be demonstrated which can possibly be attributed to the individual empowerment by strengthening self-esteem and self-confidence gained in a peer-group setting of adolescents with T1D. Considering the well-known issue that adolescents with T1D are still frequently excluded from (school/summer) camps or activities when no caregivers or medical supervisors are accompanying them, our data should support that under certain conditions, which have to be defined by the health care professionals in charge, the participation in such camps should be possible.

Overall, our data demonstrate that in an adventure camp, aiming for preparing adolescents for management of their T1D during exercise challenges, efficient and safe glycemic management is feasible. Future strategies need to be developed how the improvement in glycemic control observed throughout the camp can be sustained thereafter.

## Figures and Tables

**Table 1:** Baseline characteristics.

Characteristic	All (*n* = 14)
Age (Years)	13.1 ± 1.6
Sex (Male/Female)	8 = m/6 = f
Diabetes Duration (Years)	6.4 ± 4.4
HbA1c (%)	7.5 ± 0.9
HbA1c (mmol/mol)	58.6 ± 9.2
MDI + CGM	21% (3/14)
CSII + CGM	36% (5/14)
AID	43% (6/14)

MDI, multiple daily injections; CGM, continuous glucose monitoring; CSII, continuous subcutaneous insulin infusion; AID, automated insulin delivery.

## Data Availability

The data that support the findings of this study are available from the corresponding author, J.K.M or H.S., upon reasonable request.

## Supplementary Materials

The following supporting information can be downloaded at https://www.mdpi.com/article/10.3390/bios14090451/s1, Supplementary Table S1: SD, standard deviation; CV, coefficient of variation; IQR, interquartile range; GMI, glucose management indicator; HBGI, high blood glucose index; LBGI, low blood glucose index; MAGE, mean amplitude of glycemic excursions; TBR, time below range; TIR, time in range; TAR, time above range. Repeated measure ANOVA followed by Tukey post-hoc tests were performed for pair-wise comparison. Supplementary Table S2: MDI, multiple daily injections; CSII, continuous subcutaneous insulin infusion; AID, automated insulin delivery; CGM, continuous glucose monitoring; TBR, time below range; TIR, time in range; TAR, time above range. Only descriptive data due to small subgroup size.
