# Supplementary material for: Improved Glycemic Control during a One-Week Adventure Camp in Adolescents with Type 1 Diabetes—The DIACAMP Study
Source: Biosensors (Basel). 2024 Sep 21;14(9):451. doi: 10.3390/bios14090451 (PMC11430097; doi:10.3390/bios14090451)
Supplement: Supplementary file 1 [file biosensors-14-00451-s001.zip › biosensors-3160406-supplementary.pdf]

biosensors

MDPI

Supplementary Materials

Standardized Continuous Glucose Monitoring Metrics during a one-week Adventure Camp in Adolescents with Type 1 Diabetes – The DIACAMP Study

Improved Glycemic Control during a One-Week Adventure Camp in Adolescents with Type 1 Diabetes—The DIACAMP Study

Antonia-Therese Kietaibl [1], Faisal Aziz [2], Eva Wurm [3], Celine Tomka [2], Elke Fröhlich-Reiterer [4], Othmar Moser [2,5], Thomas R. Pieber [2], Peter Fasching [1], Julia K. Mader [2,*,†], Harald Sourij [2,6,*,†] and Felix Aberer [2]

1 Department of 5th Internal Medicine with Endocrinology, Rheumatology and Gerontology, Clinic Ottakring, 1160 Vienna, Austria; antonia-therese.kietaibl@gesundheitsverbund.at (A.T.K.); peter.fasching@gesundheitsverbund.at (P.F.)
2 Division of Endocrinology and Diabetology, Medical University of Graz, 8036 Graz, Austria; faisal.aziz@medunigraz.at (F.A.); celine.tomka@stud.medunigraz.at (C.T.); othmar.moser@medunigraz.at (O.M.); thomas.pieber@medunigraz.at (T.R.P.); felix.aberer@medunigraz.at (F.A.)
3 Division of Internal Medicine, Diakonissen Hospital Schladming, 8970 Schladming, Austria; eva.wurm@gmx.at
4 Department of Pediatrics and Adolescent Medicine, Medical University of Graz, 8036 Graz, Austria; elke.froehlich-reiterer@medunigraz.at
5 Division Exercise Physiology and Metabolism, Institute of Sport Science, University of Bayreuth, 95444 Bayreuth, Germany
6 Interdisciplinary Metabolic Medicine Trials Unit, Medical University of Graz, 8036 Graz, Austria
* Correspondence: julia.mader@medunigraz.at (J.K.M.); ha.sourij@medunigraz.at (H.S.)
† These authors contributed equally to this work.

Supplementary Table S1. SD, standard deviation; CV, coefficient of variation; IQR, interquartile range; GMI, glucose management indicator; HBGI, high blood glucose index; LBGI, low blood glucose index; MAGE, mean amplitude of glycaemic excursions; TBR, time below range; TIR, time in range; TAR, time above range. Repeated measure ANOVA followed by Tukey post-hoc tests were performed for pair-wise comparison.

	**Before camp**	**During camp**	**After camp**	**Overall P**	**P1 During vs. Before camp**	**P2 After vs. Before camp**	**P3 After vs. During camp**
**Day + Night**							
Glucose							
Mean	187.63 (40.69)	153.52 (17.65)	176.32 (27.79)	**0.001**	**0.001**	0.357	**0.024**
SD	74.84 (16.30)	58.65 (9.70)	70.47 (15.25)	**<0.001**	**<0.001**	0.352	**0.002**
CV	40.13 (5.27)	38.17 (4.46)	39.75 (4.36)	0.249	0.257	0.948	0.405
Median	175.57 (44.83)	142.75 (18.12)	163.96 (27.07)	**0.005**	**0.004**	0.429	0.073
IQR	109.04 (33.69)	72.48 (20.66)	100.05 (31.07)	**<0.001**	**<0.001**	0.530	**0.007**
Minimum	49.71 (10.91)	43.71 (7.30)	46.64 (8.78)	**<0.001**	0.198	0.640	0.666
Maximum	390.93 (20.48)	379.79 (20.34)	376.86 (32.75)	0.185	0.190	0.344	0.926
GMI	7.80 (0.97)	6.98 (0.42)	7.53 (0.66)	**0.001**	**0.001**	0.357	**0.024**
HBGI	12.74 (7.20)	6.36 (2.91)	10.41 (4.96)	**<0.001**	<0.001	0.261	**0.025**
LBGI	0.63 (0.46)	0.86 (0.44)	0.74 (0.43)	0.178	0.566	0.651	0.154
MAGE	177.21 (36.53)	147.96 (21.87)	169.33 (25.80)	**0.003**	**0.002**	0.577	**0.028**
TBR < 54 mg/dl	0.49 (0.80)	0.64 (0.49)	0.58 (0.68)	0.715	0.694	0.878	0.941
TBR < 70 mg/dl	1.82 (1.90)	3.18 (2.07)	2.59 (1.79)	**0.042**	**0.033**	0.298	0.490
TBR 54–69 mg/dl	1.33 (1.18)	2.53 (1.68)	2.01 (1.21)	**0.013**	**0.009**	0.183	0.355
TIR 70–180 mg/dl	53.12 (20.23)	70.39 (11.08)	57.65 (12.80)	**0.001**	**0.001**	0.543	**0.016**
TAR >180 mg/dl	45.06 (20.54)	26.43 (11.29)	39.76 (13.63)	**<0.001**	**0.001**	0.447	**0.013**
TAR >181–250 mg/dl	22.36 (6.11)	18.27 (6.28)	22.93 (4.62)	**0.014**	**0.043**	0.931	**0.019**
TAR >250 mg/dl	22.70 (16.45)	8.16 (6.20)	16.82 (11.93)	**<0.001**	**<0.001**	0.191	**0.036**
**Night**							
Glucose							
Mean	164.21 (47.06)	139.44 (27.22)	173.45 (35.85)	**0.003**	**0.032**	0.582	**0.003**
SD	60.56 (29.06)	45.51 (21.82)	61.25 (21.71)	**0.009**	**0.023**	0.991	**0.017**
CV	35.60 (10.46)	31.54 (8.46)	34.50 (7.31)	0.259	0.251	0.899	0.469
Median	153.82 (46.06)	131.86 (20.91)	165.39 (35.14)	**0.008**	**0.088**	0.484	**0.006**
IQR	87.96 (63.77)	61.70 (40.18)	95.00 (42.29)	**0.006**	**0.036**	0.762	**0.007**
Minimum	60.86 (12.09)	51.21 (11.48)	59.14 (14.94)	0.107	0.116	0.928	0.224
Maximum	304.36 (82.43)	270.07 (57.50)	316.00 (66.84)	0.079	0.223	0.833	0.077
GMI	7.24 (1.13)	6.65 (0.65)	7.46 (0.86)	**0.003**	**0.032**	0.582	**0.003**
HBGI	9.00 (7.91)	4.32 (4.74)	9.75 (6.28)	**0.003**	**0.013**	0.872	**0.004**
LBGI	1.01 (1.27)	1.14 (0.88)	0.68 (0.56)	0.344	0.332	0.561	0.911

MAGE	148.24 (75.87)	107.43 (65.01)	138.91 (67.57)	**0.024**	**0.025**	0.799	0.097
TBR < 54 mg/dl	1.43 (4.83)	1.15 (1.26)	0.56 (1.09)	0.691	0.959	0.688	0.835
TBR < 70 mg/dl	3.01 (6.16)	4.47 (4.41)	2.27 (2.19)	0.384	0.632	0.889	0.364
TBR 54–69 mg/dl	1.53 (1.88)	3.32 (3.36)	1.71 (1.41)	0.084	0.109	0.985	0.149
TIR 70–180 mg/dl	65.27 (24.42)	80.07 (15.52)	59.43 (20.49)	**0.002**	**0.029**	0.536	**0.002**
TAR > 180 mg/dl	31.73 (24.91)	15.46 (14.62)	38.30 (19.91)	**<0.001**	**0.015**	0.454	**0.001**
TAR >181–250 mg/dl	17.05 (12.31)	10.84 (7.63)	22.87 (11.13)	**0.002**	0.122	0.154	**0.001**
TAR > 250 mg/dl	14.68 (17.20)	4.62 (10.01)	15.43 (15.69)	**0.009**	**0.023**	0.976	**0.014**
**Day**							
Glucose							
Mean	195.10 (40.27)	158.31 (19.30)	177.28 (27.07)	**<0.001**	**<0.001**	0.095	0.072
SD	76.05 (14.27)	59.45 (8.17)	72.10 (14.44)	**0.001**	**<0.001**	0.483	**0.003**
CV	39.36 (5.14)	37.62 (3.69)	40.56 (4.54)	0.105	0.407	0.642	0.089
Median	185.07 (45.22)	147.93 (21.30)	165.07 (28.92)	**0.003**	**0.002**	0.121	0.205
IQR	112.30 (30.43)	77.70 (18.45)	101.11 (26.64)	**<0.001**	**<0.001**	0.325	**0.014**
Minimum	53.07 (13.33)	45.79 (9.93)	48.00 (10.05)	0.143	0.133	0.360	0.817
Maximum	390.93 (20.48)	375.07 (26.88)	376.57 (32.88)	0.184	0.216	0.281	0.985
GMI	7.98 (0.96)	7.10 (0.46)	7.55 (0.65)	**<0.001**	**<0.001**	0.095	0.072
HBGI	13.96 (7.30)	7.05 (3.03)	10.63 (4.87)	**<0.001**	**<0.001**	0.086	0.061
LBGI	0.55 (0.46)	0.76 (0.43)	0.76 (0.45)	0.141	0.998	0.206	0.188
MAGE	178.24 (30.89)	154.88 (20.63)	172.07 (22.59)	**0.002**	**0.002**	0.574	**0.023**
TBR < 54 mg/dl	0.40 (0.63)	0.47 (0.40)	0.59 (0.61)	0.511	0.877	0.481	0.776
TBR < 70 mg/dl	1.66 (1.89)	2.74 (1.81)	2.70 (2.03)	0.080	0.112	0.133	0.995
TBR 54–69 mg/dl	1.26 (1.33)	2.27 (1.51)	2.11 (1.49)	**0.037**	**0.044**	0.100	0.915
TIR 70–180 mg/dl	49.08 (19.11)	67.11 (11.67)	57.08 (11.91)	**<0.001**	**0.001**	0.155	0.060
TAR > 180 mg/dl	49.27 (19.58)	30.15 (12.34)	40.22 (13.20)	**<0.001**	**<0.001**	0.112	0.069
TAR >181–250 mg/dl	23.89 (5.22)	20.78 (7.04)	22.93 (4.14)	0.159	0.148	0.822	0.386
TAR > 250 mg/dl	25.37 (16.95)	9.36 (6.31)	17.29 (11.77)	**<0.001**	**<0.001**	0.064	0.070

Supplementary Table S2. MDI, multiple daily injections; CSII, continuous subcutaneous insulin infusion; AID, automated insulin delivery; CGM, continuous glucose monitoring; TBR, time below range; TIR, time in range; TAR, time above range. Only descriptive data due to small subgroup size.

	Before camp	During camp	After camp
**MDI + CGM (n=3)**			
TBR <70 mg/dl	2.78 (2.80)	4.03 (1.38)	3.71 (2.08)
TIR 70-180 mg/dl	63.48 (25.09)	69.40 (14.37)	69.13 (10.50)
TAR >180 mg/dl	33.74 (22.51)	26.57 (13.22)	27.16 (8.57)
**CSII + CGM (n=5)**			
TBR <70 mg/dl	1.42 (1.52)	3.43 (2.18)	2.30 (1.36)
TIR 70-180 mg/dl	41.66 (16.85)	65.35 (13.61)	50.46 (8.77)
TAR >180 mg/dl	56.92 (18.31)	31.22 (15.12)	47.24 (9.26)
**AID (n=6)**			
TBR <70 mg/dl	1.67 (1.92)	2.54 (2.37)	2.27 (2.03)
TIR 70-180 mg/dl	57.5 (19.15)	75.09 (6.00)	57.91 (13.77)
TAR >180 mg/dl	40.83 (19.66)	22.37 (6.01)	39.82 (15.28)
